# Relation between pulpal neuropeptides and dental caries

**Published:** 2010-08-15

**Authors:** Ali Kangarlou Haghighi, Shima Nafarzadeh, Yazdan Shantiaee, Mandana Naseri, Zohreh Ahangari

**Affiliations:** 1 *Department of Endodontics, Iranian Center for Endodontic Research, Dental School, Shahid Beheshti University of Medical Sciences, Tehran, Iran.*; 2 *Department of Oral and Maxillofacial Pathology, Dental School, Babol University of Medical Sciences, Babol, Iran.*; 3 *Department of Endodontics, Iranian Center for Endodontic Research, Dental School, Shahid Beheshti University of Medical Sciences, Tehran, Iran.*

**Keywords:** Caries, CGRP, Dental pulp, Neuropeptide

## Abstract

**INTRODUCTION:** Dental pulp has neural fibers that produce neuropeptides like Substance P (SP) and calcitonin gene-related peptide (CGRP). The inflammation of dental pulp can lead to an increase amount of SP and CGRP release, especially in symptomatic irreversible pulpitis. Therefore, it can be assumed that neuropeptides have some role in the progression of inflammation of the dental pulp. The aim of this study was to determine the relation between the presence and concentration of neuropeptides in dental pulps of carious teeth caries.

**MATERIALS AND METHODS:** For this purpose, pulpal tissues were collected from 40 teeth (20 carious and 20 intact). Pulpal samples were cultured for 72 hours. ELISA reader was used for the detection of SP and CGRP in supernatant fluids. Statistical analysis was made by Mann-Whitney *U* and Chi square tests.

**RESULTS:** SP and CGRP were present in 65% and 20% of inflamed pulpal samples, respectively and 40% and 5% of normal pulpal samples, respectively. Level of SP was significantly higher in inflamed pulp samples compared to intact pulps; however, there was no statistical difference when the other groups and neuropeptides were compared. The mean concentration of SP in normal pulps was 3.4 times greater than that of CGRP; interestingly in inflamed pulps the concentration of SP was 22.3 times greater than CGRP.

**CONCLUSION:** We can conclude that in inflamed dental pulps, the concentration of SP is higher than CGRP. It can be hypothesized that CGRP has less effect on the inflammatory changes of dental pulps.

## INTRODUCTION

Dental caries is the most common cause of the inflammation of dental pulp. It is characterized by the presence of different inflammatory leukocytes that produce a wide variety of inflammatory mediators such as cytokines and prostaglandins (1-4). It would be reasonable to assume that these sensory-derived neuropeptides have some essential role in the microvasculature of connective tissue such as vasodilation and the increase of vascular permeability. This then leads to extravasation of plasma (5,6). Calcitonin gene-related peptide (CGRP) and Substance P (SP) has been shown to enhance other aspects of immune responses, such as release histamine from mast cells, chemotaxis and phagocytosis (7,8), synthesis of other inflammatory mediators including cytokines (9-11) and metabolites of arachidonic acid (12). Their overall effect initiates the neurogenic inflammation. In response to injury, the sensory nerve fibers undergo significant changes that could be responsible for providing a local source of neuropeptides (13-16). Also, dental pulp is highly innervated vascular tissue with sensory nerve fibers which contain SP and CGRP and both have been discovered in the dental pulp (17-22). Both of these neuropeptides are thought to be major initiators of neurogenic inflammation (23).

**Figure 1:**
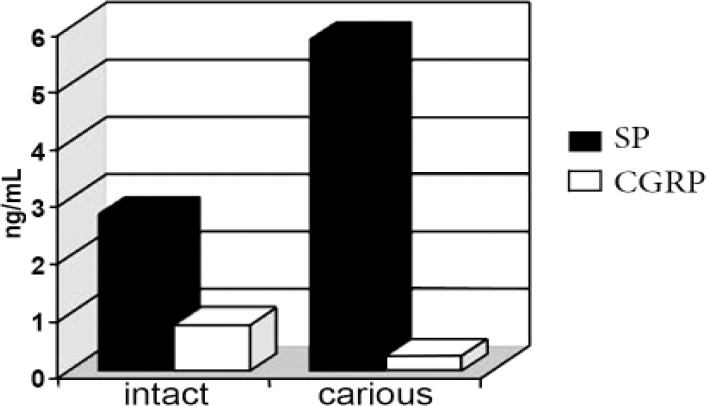
Comparison of CGRP and SP concentration (pg/mL) between intact and carious samples

Several studies have shown that SP plays a key role in the inflammatory processes of dental pulps especially in the cases of symptomatic irreversible pulpitis (24-28). In response to caries, there is an increase in neuropeptides expression in dental pulps (29,30). In addition to sensory nerve fibers, it was suggested that fibroblasts play a role in neurogenic inflammation of the pulp (31). However, studies have not analyzed and compared the presence of both SP and CGRP, between carious and noncarious dental pulp; therefore, the aim of this study was to determine the relation between pulpal SP and CGRP and caries.

## MATERIALS AND METHODS

In this case-control study was performed following the recommendations and guidelines on ethics in research with human tissues. Patients for the study were selected from Shahid Beheshti University of Medical Sciences, Dental School and signed an informed consent document approved by the Institutional Review Board.

In this case-control study, dental pulps from normal (n=20) or carious (n=20) teeth were accessed by mechanically halving the teeth immediately after extraction from forty patients. The average age of patients in case and control groups were 38.5±14.01 yrs (50% female, 50% male) and 29.4±14.01 yrs. (60% female, 40% male), respectively. Patients included in the study did not have systemic disease and had not taken antibiotic, nonsteroidal anti- inflammatory drugs, H1 and H2 antagonists in the past 3 months. In the case group, the distance of the carious lesion from the pulp chamber was more than 1 mm. Tissue samples were placed in culture medium [RPMI-1640 (10g/L) and Fetal Bovine Serum (10%)] on ice immediately after surgical excision. The samples were then rinsed with a solution of 10 g/L RPMI 1640, 100 UI/mL penicillin and 100μg/mL streptomycin. They were cut into small pieces (1 mm^3^) with a surgical scalpel in sterile Petri dishes. Each segment (corresponding to one sample) was scratched with sterile surgical blades (No.15) and then moved to a single well of a 96-well tissue culturing plate and submerged in 300 µL of a culture medium containing 10 g/L RPMI-1640, Fetal Bovine Serum, Penicillin-Streptomycin (100 UI/mL penicillin and 100 μg/mL streptomycin). As neuropeptides and inflammatory mediators cascades require some time to initiate, the traumatic splitting and cutting of tissue samples of the pulp could not induce that much influence. Moreover, even if after splitting the teeth and severing pulp tissue following extraction there were some inflammatory changes, these would occur equally in both groups.

**Figure 2:**
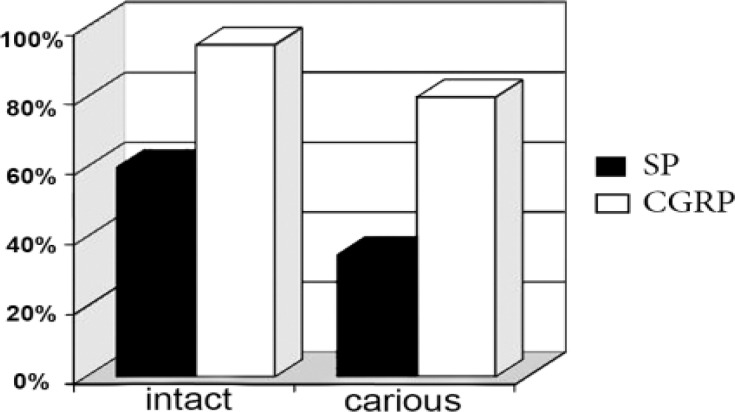
Comparison of CGRP and SP presence (%) between intact and carious samples

The plates were then transferred to an incubator with 5% CO_2_ and kept for 72 hours. Tissue culture medium was replenished at 24, 48, and 72 hours. Subsequent to each time interval, the supernatant medium was extracted with tuberculin syringes and frozen at -70˚C. Histopathologic assessment verified the viability of cells after culturing.

In order to determine the presence and concentration of CGRP (CM589101 Human CGRP ELISA kit, IBL, Germany) and SP (CM583751 Human SP ELISA kit, IBL, Germany) in supernatant fluids, ELISA method was used. Statistical analysis was conducted with Mann-Whitney *U* and Chi Square tests.

## RESULTS

CGRP was found in 5% (mean concentration of 0.80±3.57 ng/mL) and 20% (mean concentration of 5.80±5.70 ng/mL) of intact and carious samples, respectively. SP was present in 40% (mean concentration of 2.75±3.81 ng/mL) and 60% (mean concentration of 5.80±5.70 ng/mL) of intact and carious samples, respectively. There was a significant difference in the level of SP between case and control groups (P<0.05; exact value   0.032), but there was no significant difference between SP and CGRP concentration in either intact or carious samples. In Figures 1 and 2 the concentration and the presence of CGRP and SP between case and control groups have been compared with each other. There was no significant correlation between SP and CGRP based on Spearman’s correlation coefficient.

## DISCUSSION

The concentration of SP in normal pulps was 3.4 times greater than CGRP and in the inflamed pulps the concentration of SP was 22.3 times greater. However, due to difference in their rate of production and roles, we could not statistically compare SP and CGRP with each other.

We found a significant difference between carious and intact samples SP concentrations. In response to caries, there is an increase in SP expression, with greater SP expression 8 in symptomatic teeth (30). Others have reported that fibroblasts of carious teeth produce greater levels of SP compared with normal teeth (31). Results from the present study are consistent with the above studies. Substance P has been shown to stimulate monocytes-macrophages to produce tumor necrosis factor-alpha, an important inflammatory cytokine (10). SP therefore has an important role in inflammation. Reports have suggested that pulpal sensory nerve fibers and their products may have an influence upon the immune defense of the dental pulp (32). We found higher amounts of both SP and CGRP (CGRP not significant) in carious compared with normal samples. This indicates that SP may be involved in the inflammatory process of dental pulp via the sensory nerve fibers. This may be due to higher production rate of SP neuropeptides and also its role in inflammatory process. Interestingly, there is some evidence indicating that CGRP has some anti-inflammatory roles.

A study found that morphological association with calcitonin gene-related peptide immunoreactive nerve fibers, the edges of healing pulpal lesions and zones of reparative dentin. This suggests that these fibers and CGRP are involved in the healing response of pulpal tissue (33). A report also suggested a possible role for CGRP and even SP in wound healing in dental pulp (34). The mean extracellular levels of SP were significantly higher (>8-fold) in teeth diagnosed with irreversible pulpitis than SP levels in pulps diagnosed as normal (28). Although we evaluated reversible pulpitis ather than irreversible, we found similar results to the above study. Moreover, the difference between irreversible and reversible pulpitis is becoming less distinct as the most current research trend is to treat irreversible pulpitis with pulpotomy using regenerative biomaterials.

## CONCLUSION

According to the present study, the concentration of SP is greater than CGRP in the presence of inflammation in dental pulps. Higher levels of of SP compared to CGRP could provide further evidence that CGRP has little effect on the inflammatory changes of the dental pulps and is involved in the reparative rather than inflammatory process of dental pulp.

Further in depth studies on dental pulp at different stages of inflammation are required to reach more definitive results.
